# Dual roles of *Anisakis pegreffii* proteins in macrophage immune dynamics

**DOI:** 10.3389/fimmu.2025.1595093

**Published:** 2025-06-09

**Authors:** Min-hao Zeng, Sarah Alsobaie, Xiao-xu Wang, Shan Li, Fahad M. Aldakheel, Mourad A. M. Aboul-Soud, Wei-bin Liu

**Affiliations:** ^1^ Zhejiang Provincial Key Laboratory of Aquatic Resources Conservation and Development, College of Life Science, Huzhou University, Huzhou, China; ^2^ School of Biotechnology, Jiangsu University of Science and Technology, Zhenjiang, China; ^3^ Precision Preventive Medicine Laboratory of Basic Medical School, Jiujiang University, Jiujiang, Jiangxi, China; ^4^ Department of Clinical Laboratory Sciences, College of Applied Medical Sciences, King Saud University, Riyadh, Saudi Arabia; ^5^ Center of Excellence in Biotechnology Research, College of Applied Medical Sciences, King Saud University, Riyadh, Saudi Arabia

**Keywords:** *Ansiakis pegreffii*, glycoproteins, macrophage polarization, inflammation, transcriptomics

## Abstract

**Background:**

Anisakis infections have become a significant public health concern primarily caused by consuming raw or undercooked seafood. This in is due to a shift in global eating habits, and seafood consumption is becoming increasingly popular.

**Materials and Methods:**

This study explores how *Anisakis pegreffii* body proteins (ABP) and glycoproteins affect macrophage polarization. The parasites collected from East China Sea hairtail fish (*Trichiurus lepturus*), with glycoproteins isolated from ABP via ConA magnetic beads. RAW264.7 macrophages were treated with ABP, glycoproteins, and co-incubated with LPS or IL-4, then analyzed by qPCR for TNF-α and Arg-1. Transcriptomic profiling and bioinformatics analyses also helped identify differentially expressed genes and pathways.

**Results:**

This study showed that ABP with LPS greatly upregulated TNF-α, boosting inflammation. Conversely, glycoproteins suppressed TNF-α transcription and reduced IL-4-induced Arg-1 expression, displaying immunosuppression. Transcriptomics analysis found that ABP enriched TNF and hematopoietic pathways, with IL6 and IL1β as key pro-inflammatory genes. Glycoproteins activated cytokine-cytokine receptor interactions and hampered leukocyte migration by downregulating Ccl2 and H3c7. Notably, ABP and glycoproteins differentially regulated the JAK-STAT pathway.

**Conclusion:**

This study shows that *A. pegreffii* induces a dual-pronged immune response: ABP exacerbates inflammation, while glycoproteins suppress it. This highlights glycoproteins’ potential as therapeutic targets for modulating parasitic immunopathology and inflammatory diseases. The analysis of ABP and glycoprotein - induced immune responses provides key insights into Anisakiasis pathogenesis and may help develop new treatments.

## 1 Introduction

The consumption of seafood has exhibited a substantial surge, concomitant with socioeconomic development and a growing societal focus on health-conscious dietary practices (1, 2). Marine-derived foods are characterized by high concentrations of quality proteins, vitamins, and essential minerals, along with low lipid content and improved digestibility, aligning with contemporary dietary consumption patterns (3). However, many countries and regions worldwide have a tradition of consuming raw aquatic products. In addition to food safety concerns related to heavy metals, pathogenic microorganisms, and drug residues, foodborne parasitic contamination also poses a significant risk (4–6). The Food and Agriculture Organization of the United Nations (FAO) has identified 10 important zoonotic parasites associated with fish and seafood, and designated parasites as the primary biological hazard in aquatic products. Among these, Anisakis is a nematode that primarily parasitizes marine fish (7). The genus *Anisakis* belongs to the family Anisakidae, which is part of the superfamily Ascaridoidea. In a broad sense, Anisakis also includes the genus *Hysterothylacium*. Anisakis is globally prevalent and has become a significant health issue, especially in countries and regions such as Japan, South Korea, China, Taiwan, Portugal, and Chile, where there is a cultural tradition of consuming raw or lightly processed seafood (8). Consumption of raw or undercooked seafood contaminated with Anisakis larvae can lead to infection, resulting in severe symptoms such as intense abdominal pain, nausea, and vomiting. Additionally, allergic reactions ranging from urticaria to anaphylactic shock may also occur (9). The surface of parasitic worms and their excretory-secretory antigens are rich in glycoproteins. The complex glycan structures are considered primary candidates for host-parasite recognition events throughout all stages of parasitic infection (7). However, interactions between *Anisakis* glycoproteins and the host have not yet been reported.

With the increasing consumption of seafood, the potential public health risks associated with Anisakis infections are also increasing. However, these risks have not garnered sufficient attention. Investigating the interactions between Anisakis glycoproteins and host cells is highly significant, as these interactions provides a foundation for in-depth studies on the pathogenic mechanisms of Anisakis infections.

## 2 Materials and methods

### 2.1 Parasite collection

The worms were collected from hairtail fish (*Trichiurus lepturus*) sourced from the East China Sea near Shanghai, China. Commercially available hairtail fish were purchased from the market, and the abdominal cavity, digestive tract, and gonads of these fish were carefully examined under white light. The fish flesh was carefully excised to isolate the parasitic worms. Tissue adhering to the surface of the worms was removed, and the worms were washed three times with saline solution. The live worms were temporarily stored in saline solution and promptly processed in the subsequent steps. The retrieved nematodes were subjected to morphological and molecular biological analyses, which confirmed their identification as *Anisakis pegreffii*. The methodologies employed were consistent with those described in prior studies (8).

### 2.2 Extraction of Worm protein and glycoproteins

Five *A. pegreffii* were taken and put in a 1.5 mL centrifuge tube and 500 μL pre-cooled Phosphate buffer saline, and pre-cooled grinding beads were added into the tube. The tube was placed in the pre-cooled grinding module. Homogenized with high-speed tissue grinder, under the following condition: run for 5 seconds, pause for 10 seconds, at a frequency of 60Hz, for a total of 10 cycles. Centrifuge the homogenized sample at 4°C and 12 000×g for 10 minutes. After homogenization, the supernatant was carefully transferred to a new tube and a protease inhibitor (Merck, Germany) was added. Filter the protein solution using a 0.22 μm filter (Sartorius, Germany). The protein concentration was measured using BCA protein concentration assay kit (Beyotime, China).

Glycoproteins were isolated from the nematode proteins using canavalin A magnetic beads (Beyotime, China), following the manufacturer’s instructions. In brief, 50 µL of magnetic bead suspension was collected and mixed with 500 µL of Binding Buffer (20 mM Hepes pH 7.4, 1 mM MgCl_2_, 1 mM MnCl_2_, 1 mM CaCl_2_). The beads were then resuspended in the Binding buffer. Subsequently, 50 µg of nematode protein was added, and the mixture was incubated for 30 minutes in a rotating incubator. The magnetic beads were washed three times with 500 µL of Washing Buffer (20 mM Hepes pH 7.4, 1 mM MgCl_2_, 1 mM MnCl_2_, 1 mM CaCl_2_, 0.1% Tween-20). After discarding the supernatant using a separation rack, 50 µL of Elution Buffer (5 mM Tris pH 8.0, 150 mM NaCl, 2 M glucose) was added to elute the glycoproteins, which were then collected and re-measured the concentration. The extracted nematode proteins and purified glycoproteins were analyzed by sliver staining, the methods for which are detailed in **Supplementary 1**.

### 2.3 Incubation of RAW264.7 with worm protein

A total of 10^5^ RAW264.7 cells were transferred into 1 mL of DMEM complete culture medium (90% DMEM high-glucose medium, 10% fetal bovine serum, 1% Penicillin-Streptomycin Double Antibiotic). The cell culture flasks were maintained in an incubator at 37°C with 5% CO_2_. When the confluence reached 80%, the cells were treated with solutions containing different components, with three replicates per treatment group. The different treatment methods are as follows: Mock; Group EB, Elution Buffer; Group G, 10 µg/mL glycoproteins; Group LPS, 100 ng/mL Lipopolysaccharide (Beyotime, China); Group L + G, 100 ng/mL LPS and 10 µg/mL glycoproteins; Group ABP, 10 µg/mL Anisakis body protein; Group L + A, 100 ng/mL LPS and 10 µg/mL ABP; Group IL-4, 20 ng/mL Interleukin-4 (Beyotime, China); Group IL-4 + G, 20 ng/mL IL-4 and 10 µg/mL glycoproteins; Group IL-4 + ABP, 20 ng/mL IL-4 and 10 µg/mL ABP.

After a 24 hour incubation period, the cells were washed and collected. Total RNA was extracted using TRIzol reagent (Invitrogen, USA). The RNA was then reverse transcribed into cDNA using Hifair AdvanceFast 1st Strand cDNA Synthesis Kit (Yeasen, China) according to the manufacturer’s instructions. The remaining RNA was stored at −80°C. The transcriptional levels of TNF-α and Arg-1 in the cells subjected to different treatments were measured using real - time qPCR with ChamQ SYBR qPCR Master Mix (Vazyme, China). The Primers used are listed in **Table 1**.

**Table 1:** Primers used in this study.

Gene	Primers	Sequence (5’ - 3’)
TNF-α	TNF-F	AAC TAG TGG TGC CAG CCG AT
	TNF-R	CTT CAC AGA GCA ATG ACT CC
Arg-1	Arg-F	AGC CAG GGA CTG ACT ACC T
	Arg-R	TTG GGA GGA GAA GGC GTT TG
GAPDH (reference gene)	GAPDH-F	GTG GCA AAG TGG AGA TTG TTG
	GAPDH-R	CGT TGA ATT TGC CGT GAG TG

### 2.4 Transcriptomics analysis

RNA from Group ABP, G, and Mock were preserved in dry ice and sent to Wuhan Wanmo Technology Co., Ltd. for high-throughput sequencing using the BGI-seq 500 platform. Poor quality bases in the paired-end sequence data were trimmed and filtered using the software fastp (10). The parameters were set as follows: overrepresentation analysis was enabled, Overrepresentation sampling=50, qualified quality Phred=30, and low complexity filter was enabled. The software Salmon (v0.14.1) was used to build an index for the mouse genome (Mus musculus, GRCm39) and to perform quantitative analysis of transcripts for each sample. Differential analysis of the transcripts was performed using the R packages edgeR (version 4.0.16) and DESeq2 (version 1.42.1), with the parameters for differential gene expression set to FoldChange > |2.5| and p-value < 0.001. The online analysis tool “Weishengxin” (https://www.bioinformatics.com.cn/) was utilized to perform GO (Gene Ontology) and KEGG (Kyoto Encyclopedia of Genes and Genomes) enrichment analysis of the differentially expressed genes (DEGs) (11, 12). PPI (protein-protein interactions) networks were constructed using the online functional protein association network analysis tool STRING (https://cn.string-db.org/). Subsequently, key hub genes were identified based on their connectivity and centrality within the PPI network.

## 3 Results

### 3.1 Sliver staining assays for worm protein

**Figure 1:**
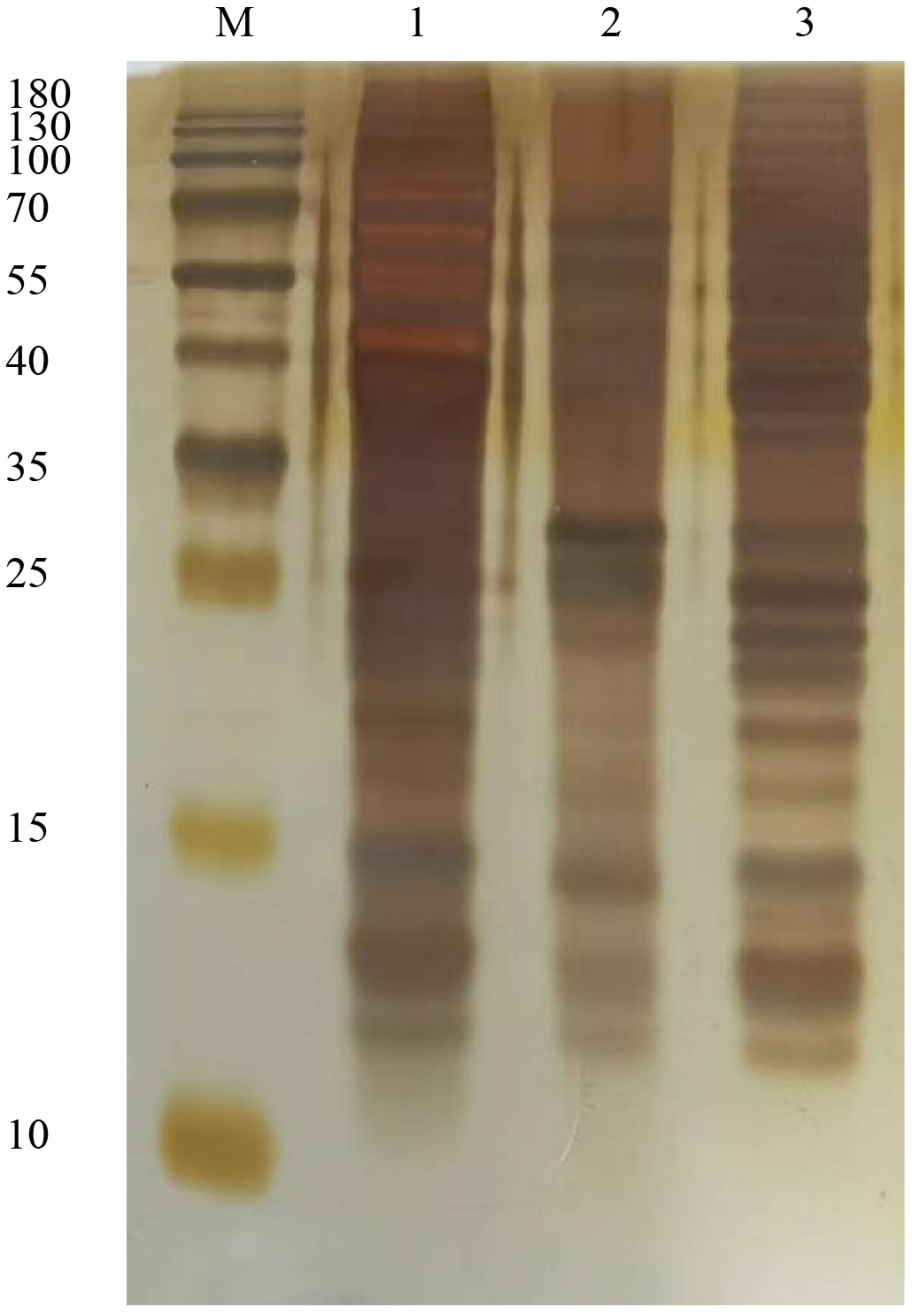
Sliver staining assay for ABP and conA combined glycoproteins. M, Marker; 1, ABP; 2, glycoproteins; 3, ABP without glycoproteins.

Purified ABP, glycoproteins, and glycoprotein depleted ABP were each loaded at 1 µg for SDS-PAGE analysis. The separated proteins were then subjected to silver staining, with the results shown in **Figure 1**. The band patterns formed by the three types of protein samples exhibited notable differences. The main band distributions of ABP and glycoprotein-depleted ABP were relatively similar. Although the glycoproteins in ABP that bind to canavalin A spanned a wide molecular weight range from 10 to over 180 kDa, the primary bands were concentrated in the 40–70 kDa and 25 kDa regions. The bands in the 10–15 kDa region were similar to those of the other two protein components, but their abundance was significantly different.

### 3.2 ABP and glycoproteins induce different macrophage polarization

**Figure 2:**
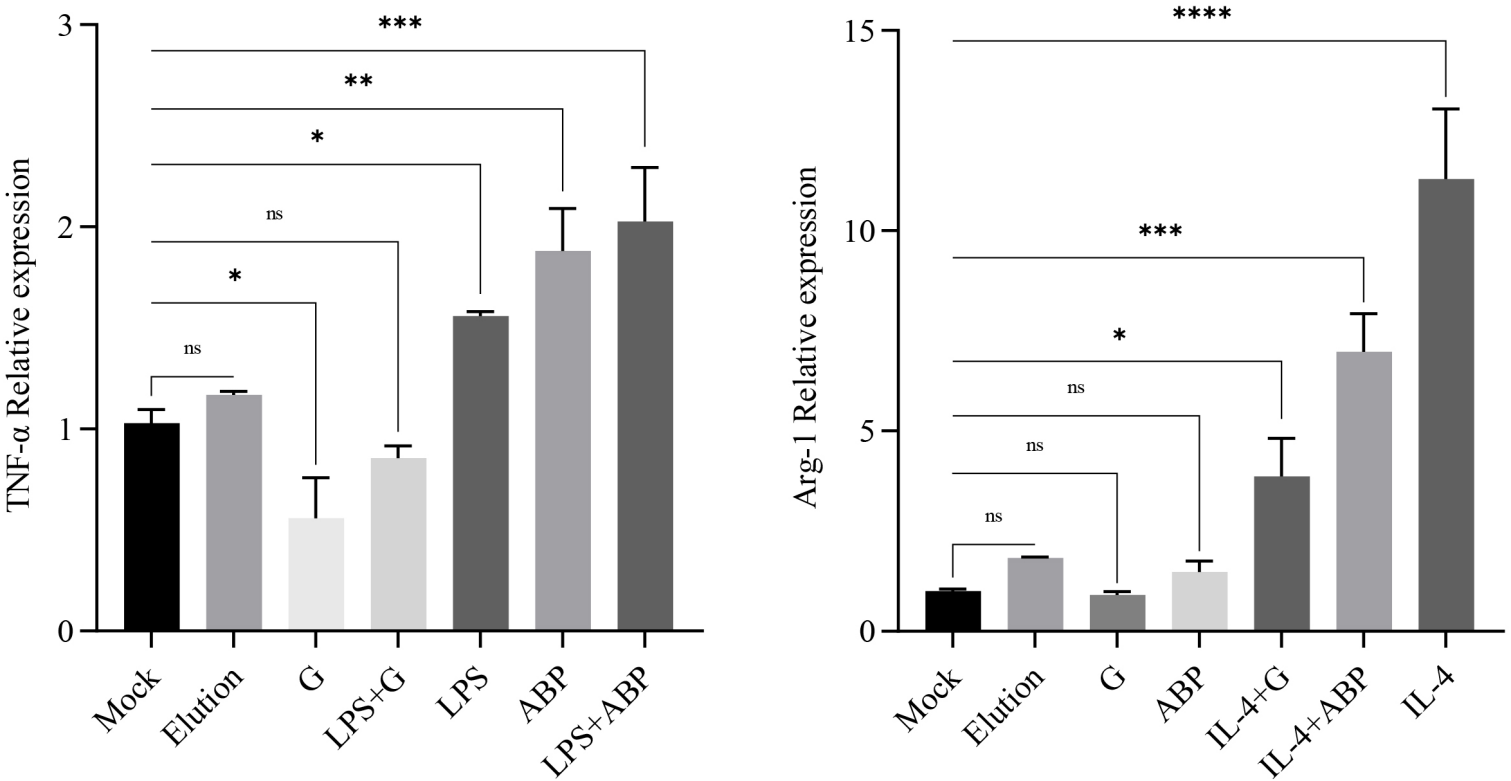
Quantitative PCR assays for the relative expression levels of TNF and Arg-1 after different component treatments. ns: p-value > 0.05; *: p-value < 0.05; **: p-value < 0.01; ***: p-value < 0.001; ****: p-value < 0.0001.

RNA was extracted from RAW264.7 cells subjected to different treatments, and RT-qPCR analysis was performed to assess the transcription levels of TNF-α and Arg-1 genes, which serve as polarization markers. The transcription levels of TNF-α and Arg-1 genes in RAW264.7 cells treated with Elution buffer did not show significant differences in comparison with the Mock group. As anticipated, LPS significantly increased the transcriptional level of TNF-α (foldchange 1.48), and the induction effect of ABP was higher than that of LPS (foldchange 1.83), with the most pronounced induction observed in cells co-incubated with both ABP and LPS (foldchange 1.97). Conversely, glycoprotein significantly inhibited the transcription of TNF-α, resulting in lower expression levels compared to the Mock group (foldchange 0.54). For the Arg-1 gene, no significant differences were observed among the Elution buffer, glycoprotein, and ABP groups when compared with the Mock group. In RAW264.7 cells co-incubated with IL-4 and either glycoprotein or ABP, the transcription of Arg-1 remained significantly higher than that in the Mock group (foldchange 4.84 and 8.73) but was significantly reduced compared with IL-4 induction alone (foldchange 14.13), with a more pronounced decrease observed in cells co-incubated with glycoprotein (**Figure 2**).

### 3.3 Transcriptome analysis

#### 3.3.1 Sequencing quality assessment

**Figure 3:**
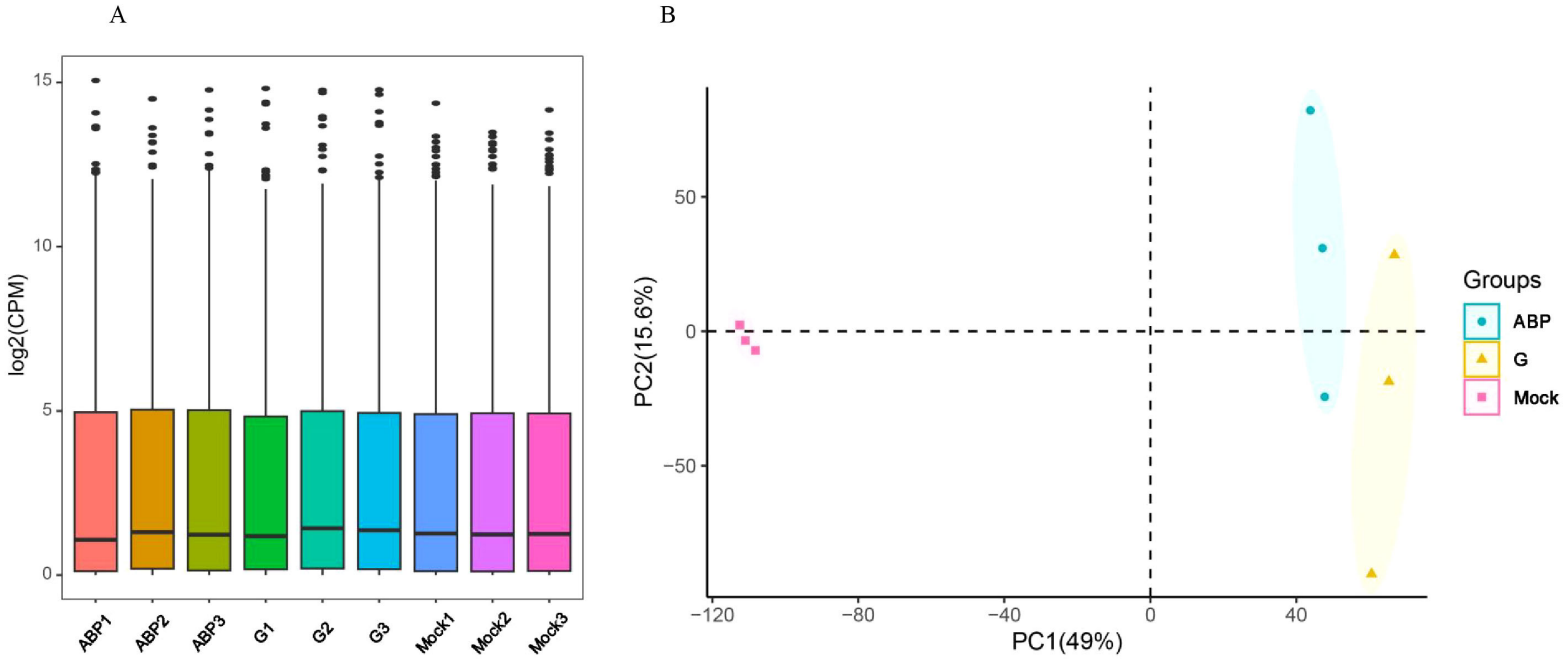
Overall transcription levels **(A)** and PCA Analysis **(B)** of Different Samples.

For each sample, the RNA sequencing generated over 43 million reads and 6.5 gigabases of data, with the Q20 and Q30 values exceeding 98% and 96%, respectively. The GC content averaged around 50% (ranging from 49.3% to 51.8%), as detailed in **Supplementary Table S1**. These data quality metrics meet the standards required for transcriptome analysis. Genes with low expression levels were filtered out based on the CPM (Counts Per Million). The overall expression distribution across samples showed minimal differences (**Figure 3A**). Principal Component Analysis (PCA) showed significant differences among the different groups, especially with the Mock group being distinctly separated from the ABP and G groups. Although the ABP group and the G group are relatively close, notable differences still exist between the two groups (**Figure 3B**).

#### 3.3.2 Identification for DEGs

**Figure 4:**
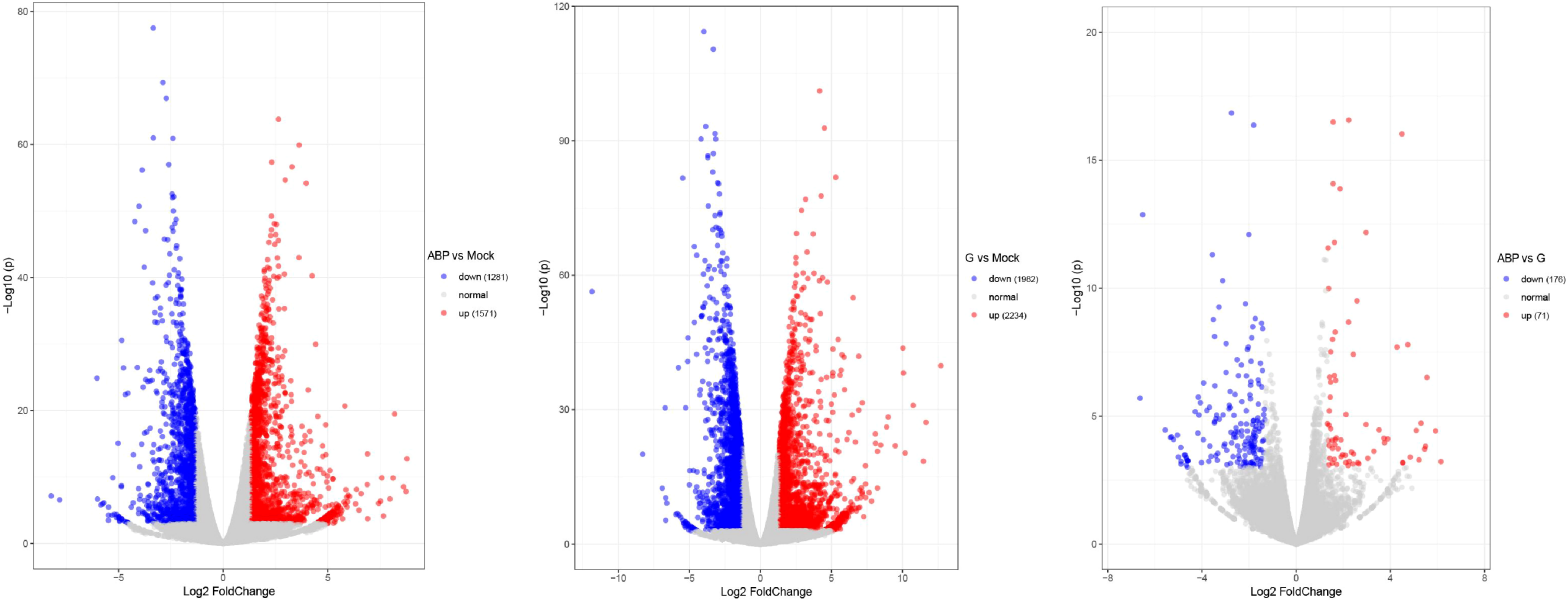
Volcano plot of differentially expressed genes in different comparison groups.

DEGs were identified by comparing the ABP group with the Mock group, the G group with the Mock group, and the ABP group with the G group (**Figure 4**). The number of DEGs vary among these comparison groups. When comparing the ABP group to the Mock group, there are 1571 upregulated genes and 1281 downregulated genes. In contrast, the G group exhibits a greater number of differentially expressed genes, with 2234 upregulated and 1982 downregulated genes. When comparing group ABP to Group G, Group ABP has fewer differentially expressed genes, with 71 genes upregulated and 176 downregulated genes. The DEGs are listed in **Table 2** (Top 10).

**Table 2:** DEGs in different comparative group (Top 10).

Comparative group	Gene name	Log2 FoldChange	P-value
ABP vs Mock	Tm9sf5	8.782816	1.81E-13
	Scn11a	8.73973	1.51E-08
	Clca2	8.621777	2.98E-09
	H2ac19	-8.2177	7.09E-08
	Gm43064	8.191707	3.33E-20
	Rorc	8.118566	1.41E-10
	Gm49450	7.97004	1.98E-07
	Gm2446	-7.81254	2.77E-07
	Gm2223	7.659632	7.53E-05
	Gm47095	7.578574	1.37E-10
G vs Mock	Clca2	12.69781	1.53E-40
	Gm8203	-11.8472	4.33E-57
	Scn11a	11.65562	6.88E-28
	Meox2	11.47051	3.23E-19
	Tm9sf5	10.75448	1.05E-31
	Fam20a	10.182	4.63E-21
	Gm2223	10.0676	6.3E-39
	Gm47095	10.03362	1.79E-44
	Rorc	9.48667	1.14E-22
	Prss35	8.992979	4.3E-29
ABP vs G	Gm8203	10.78211024	8.79464E-35
	Gm2446	-9.30097731	1.16801E-08
	H2ac19	-8.130389346	7.01903E-16
	Krt23	-6.627429331	1.97976E-06
	Cryab	-6.515247469	1.34906E-13
	Pet117	6.146815262	0.000601494
	Gm12397	5.915107743	3.77065E-05
	Prl2c3	-5.561638017	3.47624E-05
	Gm11578	5.553595797	3.08499E-07
	Gm33103	5.48985597	0.000150209

#### 3.3.3 GO function analysis

**Figure 5:**
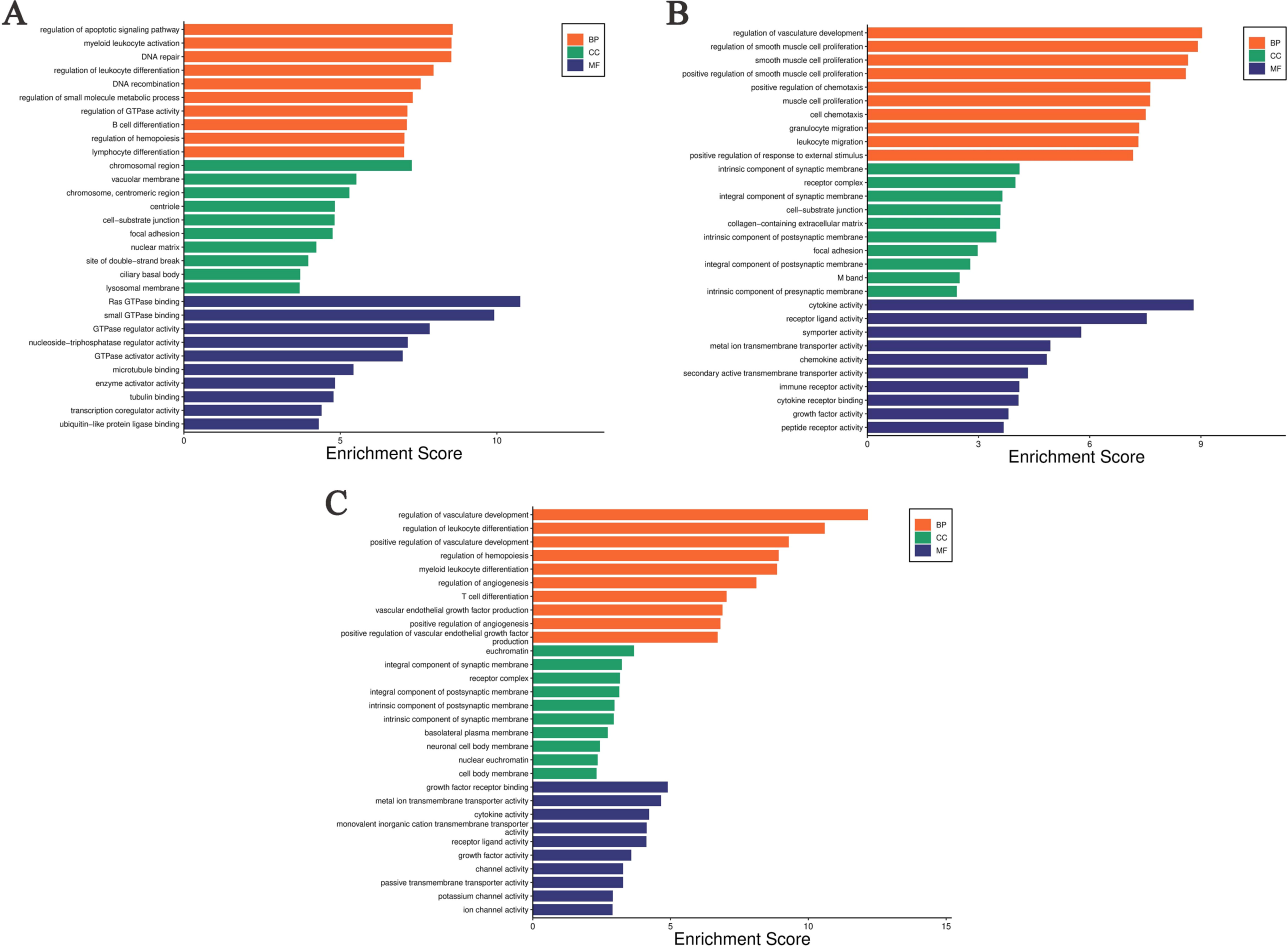
Gene Ontology enrichment analysis of differentially expressed genes in different comparison groups. **(A)** ABP VS Mock group; **(B)** G VS Mock group; **(C)** ABP VS G group.

Performs GO enrichment analysis on DEGs across various comparison groups. The DEGs identified between the ABP group, and the Mock group show significant enrichment at the Biological Process (BP) level in the regulation of the apoptotic signaling pathway, myeloid leukocyte activation, DNA repair, regulation of leukocyte differentiation, DNA recombination, regulation of small molecule metabolic process, regulation of GTPase activity, B cell differentiation, regulation of hematopoiesis, and lymphocyte differentiation. At the Cellular Component (CC) level, enrichment was observed in chromosomal regions, vacuolar membranes, centromeric regions of chromosomes, centrioles, cell-substrate junctions, focal adhesions, the nuclear matrix, site of double-strand breaks, ciliary basal bodies, and lysosomal membrane. At the Molecular Function (MF) level, enrichment is observed in Ras-GTPase binding, small GTPase binding, GTPase regulatory activity, nucleoside triphosphate regulatory activity, GTPase activator activity, microtubule binding, enzyme activator activity, tubule binding, transcription coregulator activity, and ubiquitin-like protein binding (**Figure 5A**).

The DEGs between the G group and the Mock group are significantly enriched at the BP level in several areas, including the regulation of vasculature development, r muscle proliferation-related processes, positive regulation of chemotaxis, cell chemotaxis, granulocyte migration, leukocyte migration, and positive regulation of response to external stimulus. At the CC level, these DEGs are enriched in intrinsic components of synaptic membrane, receptor complexes, synaptic membrane components, cell-substrate junctions, collagen-containing extracellular matrix, intrinsic components of postsynaptic membrane, focal adhesions, intrinsic components of postsynaptic density, M bands, and intrinsic components of presynaptic membrane. At the MF level, they are enriched in cytokine activity, receptor ligand activity, symporter activity, metal ion transmembrane transporter activity, chemokine activity, secondary active transmembrane transporter activity, immune receptor activity, cytokine receptor activity, growth factor activity, and peptide receptor activity (**Figure 5B**).

The differentially expressed genes between the ABP group and the G group are enriched at the BP level in the processes related with vascular development, regulation of leukocyte differentiation, the processes related with hematopoiesis, myeloid leukocyte differentiation, regulation of angiogenesis, regulation of T-cell differentiation. The differentially expressed genes are enriched at the CC level in euchromatin, synaptic membrane components, postsynaptic membrane components, receptor complexes, postsynaptic membrane parts, intrinsic components of synaptic membrane, basolateral plasma membrane, neuronal cell body membrane, nuclear chromatin, and somatic membrane. At the MF level, the enrichment is observed in growth factor receptor binding, metal ion transmembrane transporter activity, cytokine activity, monovalent cation transmembrane transporter activity, receptor ligand activity, growth factor activity, channel activity, passive transmembrane transporter activity, potassium channel activity, and ion channel activity (**Figure 5C**).

#### 3.3.4 KEGG annotation

**Figure 6:**
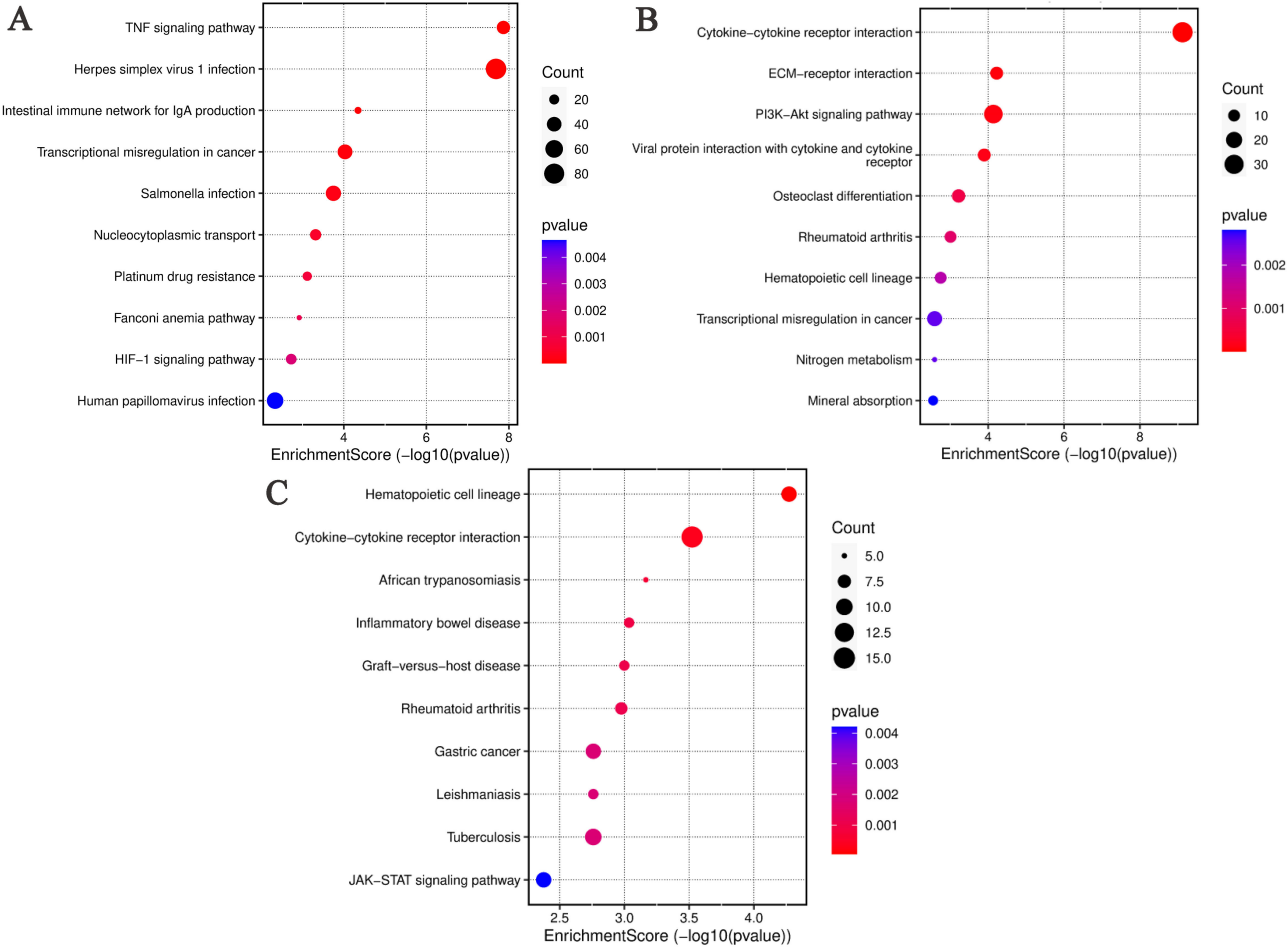
KEGG analysis of differentially expressed genes in different comparison groups. **(A)** ABP VS Mock group; **(B)** G VS Mock group; **(C)** ABP VS G group.

The DEGs between the ABP group and the Mock group are shown significant enrichment in several KEGG pathways. These include TNF signaling pathway, Herpes simplex virus 1 infection, Intestinal immune network for IgA production, Transcriptional misregulation in cancer, Salmonella infection, Nucleocytoplasmic transport, Platinum drug resistance, Fanconi anemia pathway, HIF-1 signaling pathway, Human papillomavirus infection (**Figure 6A**).

Similarly, DEGs identified in the ABP versus Mock comparison, were associated with various pathways, such Cytokine-cytokine receptor interaction, ECM-receptor interaction, PI3K-Akt signaling pathway, Viral protein interaction with cytokine and their receptor, Osteoclast differentiation, Rheumtoid arthritis, Hematopoietic cell lineage, Transcriptional misregulation in cancer, Nitrogen metabolism, Mineral absorption (**Figure 6B**).

In the ABP versus G group comparison, DEGs were predominately enriched in pathways related to hematopoietic cell lineage, cytokine-cytokine receptor interaction, African trypanosomiasis, inflammatory bowel disease, graft-versus-host disease, rheumatoid arthritis, gastric cancer, leishmaniasis, tuberculosis, and JAK-STAT signaling pathway (**Figure 6C**).

#### 3.3.5 PPI Analysis and Identification of Key Hub Genes among DEGs

To analyze the interaction relationships among the differentially expressed genes, we used the online functional protein association network analysis tool STRING (https://cn.string-db.org/) to construct PPI (Protein-Protein Interaction) networks based on these DEGs. The central nodes of each gene in the networks are presented in **Table 3**.

**Table 3:** The nodes information of the STRING interaction network of differentially expressed genes among different groups (Top 10).

Comparative group	Node	Node degree	Log2FoldChange
ABP vs Mock	Il6	49	3.790834
	H3c8	34	-1.63833
	Il1b	32	5.815349
	Brca1	30	1.944913
	Atm	20	2.154239
	Esr1	20	2.060958
	Myc	20	-2.01265
	Cd86	17	1.793976
	Trp53bp1	17	1.765176
	Csf2	16	3.882335
G vs Mock	Il1b	34	5.472111
	Ccl2	23	-3.62871
	H3c7	22	-2.34084
	Csf3	16	3.525697
	Pdgfrb	16	2.757473
	Cxcl5	15	2.405916
	Fcgr3	15	-2.73582
	Pparg	15	-5.06256
	Ptgs2	15	5.739006
	Cxcr3	14	-3.25117
ABP vs G	Il6	26	3.751976
	H6pd	12	-1.45791
	Chac1	10	1.6615
	Fcgr3	6	1.446982
	Ifi44	6	-1.42006
	Mthfd2	6	1.566683
	Trib3	6	2.234664
	Cth	5	1.864713
	Nqo1	5	-2.6792
	Rsad2	5	-1.91419

**Figure 7:**
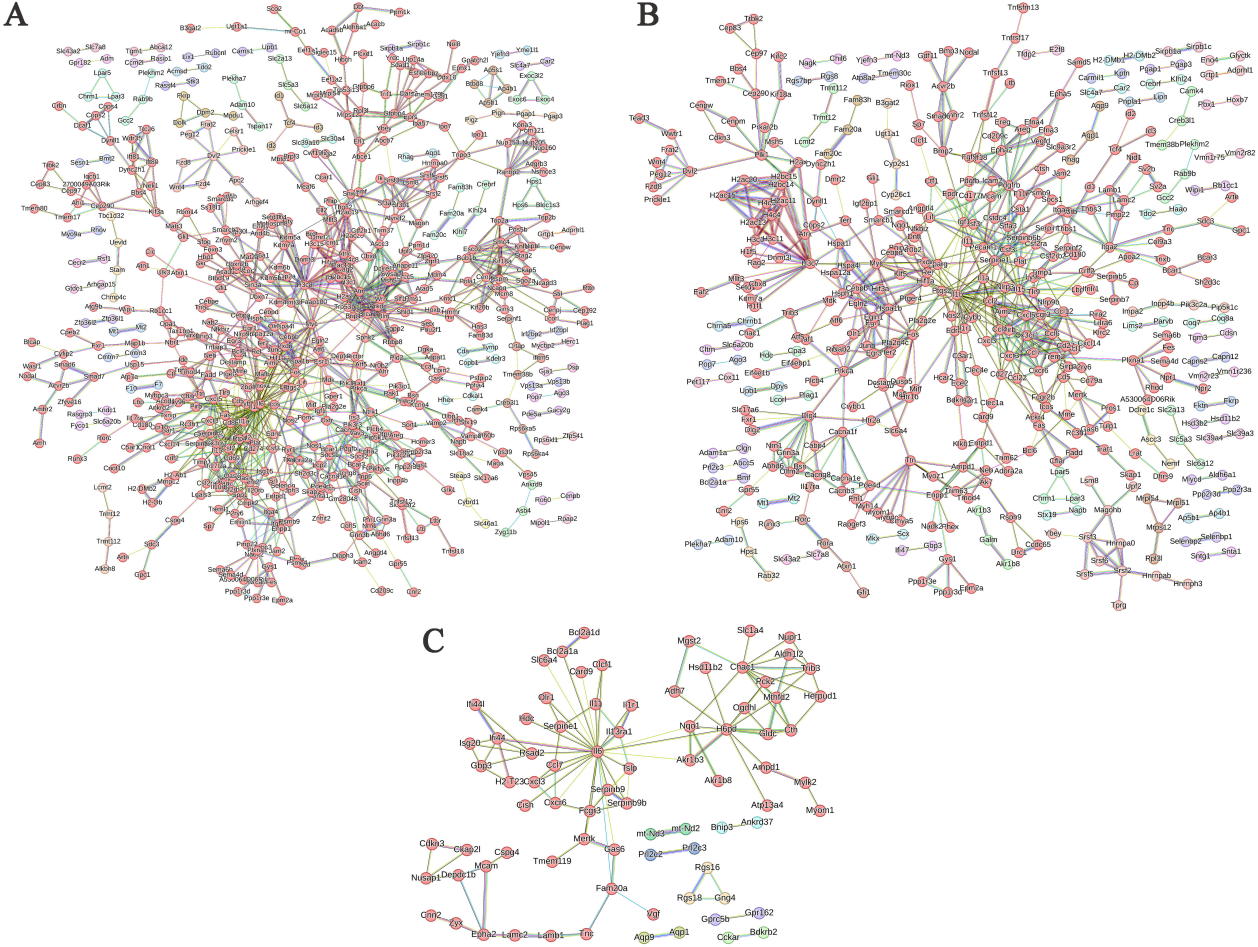
PPI network of differentially expressed genes in different comparison groups. **(A)** ABP VS Mock group; **(B)** G VS Mock group; **(C)** ABP VS G group.

In the PPI network analysis of differentially expressed genes (DEGs) between the ABP group and the Mock group, 7 genes exhibited at least 20 nodes degrees, among which 5 were upregulated and 2 were downregulated. The entire network formed three distinct gene clusters centered around IL6, H3c8, and Il1b, respectively. Notably, IL6 had the highest node degree of 49, indicating its extensive influence within the network (**Figure 7A**).

Among the DEGs identified between the G group and the Mock group, the PPI network revealed that 3 genes had node degree exceeding 20. Of these, only IL1b was upregulated, while Ccl2 and H3c7 were downregulated. The network formed two distinct gene clusters centered around IL1b and H3c8, respectively (**Figure 7B**).

The number of DEGs between the ABP group and the G group was relatively low, resulting in a simpler PPI network (**Figure 7C**). In this network, only IL6 had a node degree higher than 20, with 26 connections, and IL6 was upregulated. In contrast, H6pd and Chac1 had node degrees of 12 and 10, respectively, and both were downregulated.

## 4 Discussion

Anisakis infection is prevalent in marine fish and seafood, posing a significant threat to public health. The findings of this study indicate that ABP and the glycoproteins that bind to conA have distinct effects on macrophage polarization. The results show that ABP significantly increases the transcription of TNF-α, which is consistent with previous research, which demonstrated that human colon cells (Caco-2) exposure to Anisakis extracts *in vitro*, produce a robust inflammatory response (13). The ability of ABP to induce TNF-α transcription aligns with prior studies showing that helminth-derived proteins often trigger pro-inflammatory responses via pattern recognition receptors (PRRs) like Toll-like receptors (TLRs) or NOD-like receptors (NLRs) (14, 15). Conversely, glycoproteins suppressed TNF-α transcription to levels even below the basal levels, indicating their immunosuppressive properties. This inhibition could be mediated through glycans interacting with host lectin receptors (e.g., C-type lectins or galectins), which are known to dampen TLR signaling (16, 17). For instance, Glycoproteins from *Fasciola hepatica*, bind to mannose receptors, however, their immunosuppressive effects are not dependent on these receptors, suggesting that other types of C-type lectin receptors may be involved in their immunomodulatory mechanisms (18).

Transcriptomic profiling revealed that ABP and glycoproteins activate distinct immune pathways. Macrophages treated with ABP exhibited enrichment in TNF signaling and hematopoietic cell lineage pathways, both of which are associated with inflammation and innate immune activation. The upregulation of IL6, IL1b, and H3c8 as hub genes in the ABP group underscores their central roles in driving pro-inflammatory cascades. Previous studies have demonstrated that Anisakis proteins can up-regulate IL-6 gene expression, yet live worms and EVs (extracellular vesicles) inhibit IL-6 expression (19). Similarly, serum IL-6 levels are elevated in Anisakis - sensitized patients (20). IL6 and IL1β are pivotal cytokines in acute inflammation, and their overexpression aligns with clinical symptoms of anisakiasis, such as abdominal pain and systemic allergic reactions (21, 22).

In contrast, glycoproteins induced pathways associated with cytokine-cytokine receptor interactions, chemotaxis, and vascular development. The downregulation of Ccl2 and H3c7 in the glycoprotein group suggests impaired leukocyte migration, which may limit the host’s ability to mount the effective immune response at infection sites. Furthermore, the enrichment of the JAK-STAT signaling pathway in the comparison between ABP and glycoprotein highlights the divergent regulation of cytokine signaling, with glycoproteins potentially suppressing STAT-mediated transcriptional activation (23).

Compared to macrophages treated with glycoprotein, DEGs in the ABP group were significantly enriched in pathways related to hematopoietic cell lineage, cytokine-cytokine receptor interaction, and the inflammatory bowel disease pathways. This suggests that the differences in the effects of glycoprotein and whole-body proteins on macrophages are primarily reflected in cytokine transcription and expression. Additionally, the upregulation of key nodes such as IL6 and Chac1 in the PPI network suggests that whole-body ABP induces a more intense inflammatory response (24). The downregulation of H6pd results in a decrease in PPP intermediates, D-ribose, and NADPH levels, along with an increase in ROS, leading to oxidative stress (25).

Protein glycosylation is highly common in helminths. For instance, the genetic model organism *Caenorhabditis elegans* possesses an intricate protein glycome. The parasitic nematode *Brugia malayi* has been documented to contain 582 glycoproteins with 1,273 identified N-glycosylation sites, which may serve as potential therapeutic targets and biomarkers (26, 27). Notably, studies have revealed that glycoproteins from the intestinal parasite *Trichuris suis* can be recognized by both innate and adaptive immune systems, demonstrating species-specific immunogenicity. Furthermore, the native H11 antigen from *Haemonchus contortus* exhibits significant protective efficacy as a vaccine candidate, whereas its recombinant counterpart fails to confer similar protection. This striking contrast underscores the critical role of nematode-specific protein glycosylation in eliciting protective immune responses against parasitic helminths (28, 29).

## 5 Conclusion

This study demonstrates that *A. pegreffii* induces a dual-pronged immune response to modulate host immunity: ABP drives inflammation, while glycoproteins suppress pro-inflammatory responses. These findings advance our understanding of Anisakiasis pathogenesis and highlight glycoproteins as potential targets for therapeutic intervention. Future research should focus on delineating the molecular mechanisms of glycan-host interactions and evaluating glycoprotein-based immunomodulators for inflammatory diseases. Future research should focus on elucidating the molecular mechanisms underlying glycan-host interactions and evaluating glycoprotein-based immunomodulators for inflammatory diseases.

## Data Availability

Publicly available datasets were analyzed in this study. This data can be found here: Sequence Read Archive [https://379/www.ncbi.nlm.nih.gov/sra], BioProject ID: PRJNA1232931.
