# Correction: Dual roles of *Anisakis pegreffii* proteins in macrophage immune dynamics

**DOI:** 10.3389/fimmu.2025.1669186

**Published:** 2025-08-07

**Authors:** Min-hao Zeng, Sarah Alsobaie, Xiao-xu Wang, Shan Li, Fahad M. Aldakheel, Mourad A. M. Aboul-Soud, Wei-bin Liu

**Affiliations:** ^1^ Zhejiang Provincial Key Laboratory of Aquatic Resources Conservation and Development, College of Life Science, Huzhou University, Huzhou, China; ^2^ School of Biotechnology, Jiangsu University of Science and Technology, Zhenjiang, China; ^3^ Precision Preventive Medicine Laboratory of Basic Medical School, Jiujiang University, Jiujiang, Jiangxi, China; ^4^ Department of Clinical Laboratory Sciences, College of Applied Medical Sciences, King Saud University, Riyadh, Saudi Arabia; ^5^ Center of Excellence in Biotechnology Research, College of Applied Medical Sciences, King Saud University, Riyadh, Saudi Arabia

**Keywords:** *Ansiakis pegreffii*, glycoproteins, macrophage polarization, inflammation, transcriptomics

Adding:

Affiliation 5 “Center of Excellence in Biotechnology Research, College of Applied Medical Sciences, King Saud University, Riyadh, Saudi Arabia” was omitted for author Mourad A. M. Aboul-Soud. This affiliation has now been added for author Mourad A. M. Aboul-Soud.

Removing:

Author Mourad A. M. Aboul-Soud was erroneously assigned to affiliation 4 “Department of Clinical Laboratory Sciences, College of Applied Medical Sciences, King Saud University, Riyadh, Saudi Arabia”. This affiliation has now been removed for author Mourad A. M. Aboul-Soud.

The original version of this article has been updated.

