# Supplementary material for: Dual roles of Anisakis pegreffii proteins in macrophage immune dynamics
Source: Front Immunol. 2025 Jun 9;16:1595093. doi: 10.3389/fimmu.2025.1595093 (PMC12183258; doi:10.3389/fimmu.2025.1595093)
Supplement: Supplementary file 1 [file Table1.docx]

Supplementary Material

**Dual Roles of *Anisakis pegreffii* Proteins in Macrophage Immune Dynamics**

**Min-hao Zeng^1,2^, Sarah Alsobaie^3^, Xiao-xu Wang^2^, Shan Li^4^, Fahad M. Aldakheel^3^, Mourad A. M. Aboul-Soud^3^, Wei-bin Liu^1*^**

^1^Zhejiang Provincial Key Laboratory of Aquatic Resources Conservation and Development, College of Life Science, Huzhou University, Huzhou 313000, The People's Republic of China

^2^School of Biotechnology, Jiangsu University of Science and Technology, Zhenjiang 212100, The People's Republic of China

^3^Clinical Laboratory Sciences Department, College of Applied Medical Sciences, King Saud University, Riyadh 11433, Saudi Arabia

^4^Precision Preventive Medicine Laboratory of Basic Medical School, Jiujiang University, Jiujiang 332005, The People's Republic of China

*** Correspondence:**Wei-bin Liu
<liuweibin001301@foxmail.com>

# Supplementary Data

Supplementary Material should be uploaded separately on submission. Please include any supplementary data, figures and/or tables.

**Method of Sliver staining**

1. Add 20 mL of fixing solution (2 mL acetic acid, 8 mL methanol, and 10 mL ddH2O, which can submerge the gel) and fix for at least 1 hour on a gentle shaker.
2. After fixation, discard the fixing solution. Wash for 5 minutes on a gentle horizontal shaker with ddH2O.
3. Discard the wash solution. Add 20 mL of sensitizing solution (6 mL methanol, 0.04 g NaS2O3·5H2O, 1.36 g anhydrous sodium acetate, and ddH2O to 20 mL) and sensitize for 30 minutes on a gentle shaker.
4. Discard the sensitizing solution. Wash 3 times for 5 minutes each on a gentle horizontal shaker with ddH2O.
5. Discard the wash solution. Add 20 mL of silver staining solution (0.05 g silver nitrate and 20 mL ddH2O) and react for 20 minutes in the dark on a gentle shaker.
6. Discard the silver staining solution. Wash for 2 minutes on a gentle horizontal shaker with ddH2O.
7. Discard the wash solution. Add 20 mL of developing solution (0.5 g anhydrous sodium carbonate, 8 μL formaldehyde, and 20 mL ddH2O) and develop for 1-10 minutes with rapid shaking, monitoring the process.
8. When proper staining is achieved, discard the developing solution and add the stop solution (3.65 g EDTA and 250 mL ddH2O) to terminate the reaction.

# Supplementary Figures and Tables

For more information on Supplementary Material and for details on the different file types accepted, please see [here](https://www.frontiersin.org/guidelines/author-guidelines#supplementary-material).

## Supplementary Tables

**Supplementary Table 1.** RNA sequencing and RAW data quality in this study.

| Sample number. | Qubit  (ng/μl) | Total quantity(μg) | Reads/Bases amount | Q20 bases | Q30 bases | GC content |
| --- | --- | --- | --- | --- | --- | --- |
| Mock1 | 62.4 | 1.2 | 52.4 M / 7.8 G | 98.7% | 96.4% | 51.5% |
| Mock2 | 110 | 2.2 | 55.8 M / 8.3 G | 98.8% | 96.7% | 51.7% |
| Mock3 | 79.4 | 1.8 | 48.5 M / 7.2 G | 98.8% | 96.5% | 51.8% |
| ABP1 | 134 | 3.8 | 52.5 M / 7.8 G | 98.86% | 96.82% | 49.5% |
| ABP2 | 80.8 | 2.0 | 46.8 M / 6.9 G | 98.8% | 96.8% | 49.3% |
| ABP3 | 121 | 3.5 | 43.6 M / 6.5 G | 98.7% | 96.5% | 49.4% |
| G1 | 128 | 3.2 | 52.1 M / 7.8 G | 98.7% | 96.4% | 49.6% |
| G2 | 116 | 3.1 | 46.6 M / 6.9 G | 99.1% | 97.4% | 49.3% |
| G3 | 122 | 3.3 | 53.3 M / 7.9 G | 99.0% | 97.1% | 49.3% |
